# Supplementary material for: Deep Learning to Decipher the Progression and Morphology of Axonal Degeneration
Source: Cells. 2021 Sep 25;10(10):2539. doi: 10.3390/cells10102539 (PMC8534012; doi:10.3390/cells10102539)
Supplement: Supplementary file 1 [file cells-10-02539-s001.zip › cells-1357304 supplementary proofreading back - Copy/20210924_Palumbo et al_Cells_Supplement.pdf]

cells

Supplementary materials for Article

Deep learning to decipher the progression and morphology of axonal degeneration

Alex Palumbo [1,2,3,*], Philipp Grüning [4], Svenja Kim Landt [1,2], Lara Eleen Heckmann [1,2], Luisa Bartram [1], Alessa Pabst [1,2], Charlotte Flory [1,2], Maulana Ikhsan [1,2,3,5], Sören Pietsch [1,2,6], Reinhard Schulz [7], Christopher Kren [8], Norbert Koop [8], Johannes Boltze [1,2,9], Amir Madany Mamlouk [4] and Marietta Zille [1,2,3,10,*]

[1] Fraunhofer Research and Development Center for Marine and Cellular Biotechnology EMB, 23562 Lübeck, Germany; alex.palumbo@uni-luebeck.de (A.P.); svenja-kim.landt@gmx.net (S.K.L.); LaraEleen.Heckmann@kgu.de (L.E.H.); luisa.bartram@web.de (L.B.); apabst@ukaachen.de (A.P.); charlotte.flory@leibniz-hpi.de (C.F.); maulanaikhsan@unimal.ac.id (M.I.); soeren.pietsch@medizin.uni-leipzig.de (S.P.); johannes.boltze@warwick.ac.uk (J.B.); m.zille@uni-luebeck.de (M.Z.)
[2] Institute for Medical and Marine Biotechnology, University of Lübeck, 23562 Lübeck, Germany
[3] Institute for Experimental and Clinical Pharmacology and Toxicology, University of Lübeck, 23562 Lübeck, Germany
[4] Institute for Neuro- and Bioinformatics, University of Lübeck, 23562 Lübeck, Germany; gruening@inb.uni-luebeck.de (P.G.); madany@inb.uni-luebeck.de (A.M.M.)
[5] Faculty of Medicine, Malikussaleh University, Lhokseumawe 24355, Indonesia
[6] Department of Neonatology, Universitätsklinikum Leipzig, 04103 Leipzig, Germany
[7] Wissenschaftliche Werkstätten, University of Lübeck, 23562 Lübeck, Germany; reinhard.schulz@uni-luebeck.de
[8] Medical Laser Center Lübeck GmbH, 23562 Lübeck, Germany; christopher.kren@uni-luebeck.de (C.K.); n.koop@uni-luebeck.de (N.K.)
[9] School of Life Sciences, The University of Warwick, Gibbet Hill Campus, Coventry CV4 7AL, UK
[10] Department of Pharmaceutical Sciences, Division of Pharmacology and Toxicology, University of Vienna, 1090 Vienna, Austria; marietta.zille@univie.ac.at
* Correspondence: alex.palumbo@uni-luebeck.de, m.zille@uni-luebeck.de

Citation: Palumbo, A.; Grüning, P.; Landt, S.K.; Heckmann, L.E.; Bartram, L.; Pabst, A.; Flory, C.; Ikhsan, M.; Pietsch, S.; Schulz, R.; et al. Deep Learning to Decipher the Progression and Morphology of Axonal Degeneration. *Cells* **2021**, *10*, x. https://doi.org/10.3390/xxxxx

Academic Editor(s): Naweed I. Syed

Received: 11 August 2021
Accepted: 22 September 2021
Published: date

Publisher's Note: MDPI stays neutral with regard to jurisdictional claims in published maps and institutional affiliations.

Copyright: © 2021 by the authors. Licensee MDPI, Basel, Switzerland. This article is an open access article distributed under the terms and conditions of the Creative Commons Attribution (CC BY) license (http://creativecommons.org/licenses/by/4.0/).

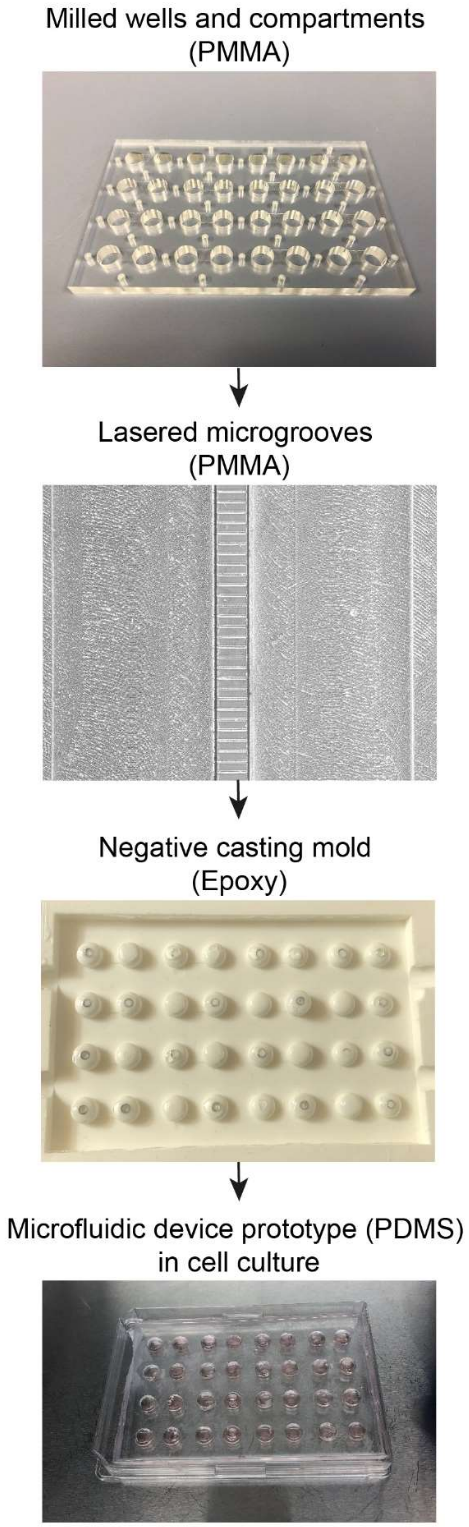

Figure S1. Manufacturing of the microfluidic device for the enhanced throughput cultivation of axons. Individual components of the production process. A milled and lasered polymethyl methacrylate (PMMA) plate was used as a positive imprint to develop a negative casting mold produced from epoxy. Polydimethylsiloxane (PDMS) was poured into the negative casting mold, bonded onto glass and applied in the cell culture.

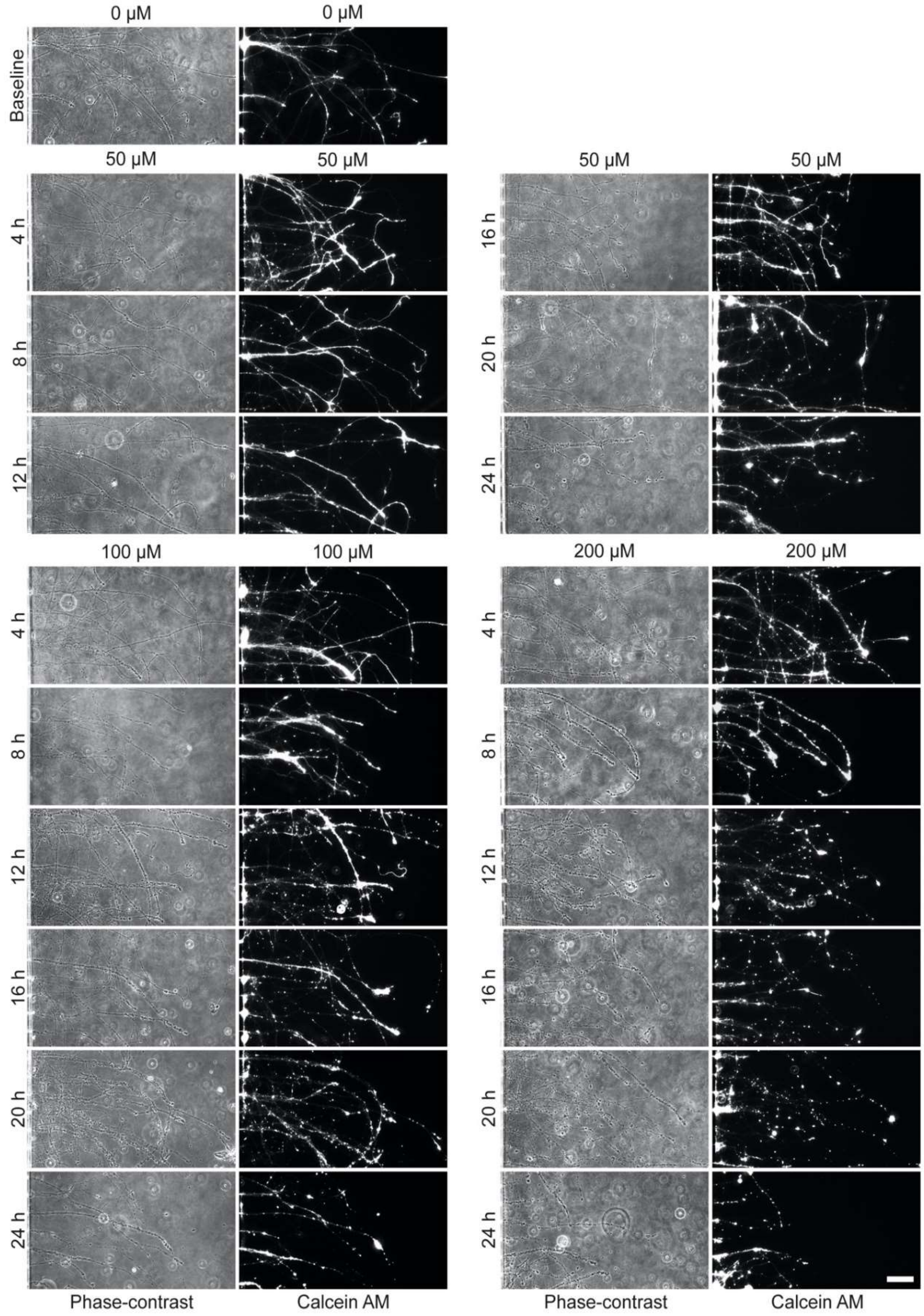

Figure S2. Validation of the time course of AxD by fluorescent live cell marker calein AM. We determined the start and end of hemin-induced AxD by comparing the appearance of axonal swellings and axonal fragments in phase-contrast and fluorescence (calcein AM) microscopy. Compared to baseline (0 μM hemin), morphological hallmarks of AxD started to appear after 16 hours (50 μM), 12 hours (100 μM), and 8 hours (200 μM) and AxD was observed during the following 4 hours in all concentrations. N = 3 independent cultures of primary cortical neurons. Scale bar: 50 μm.

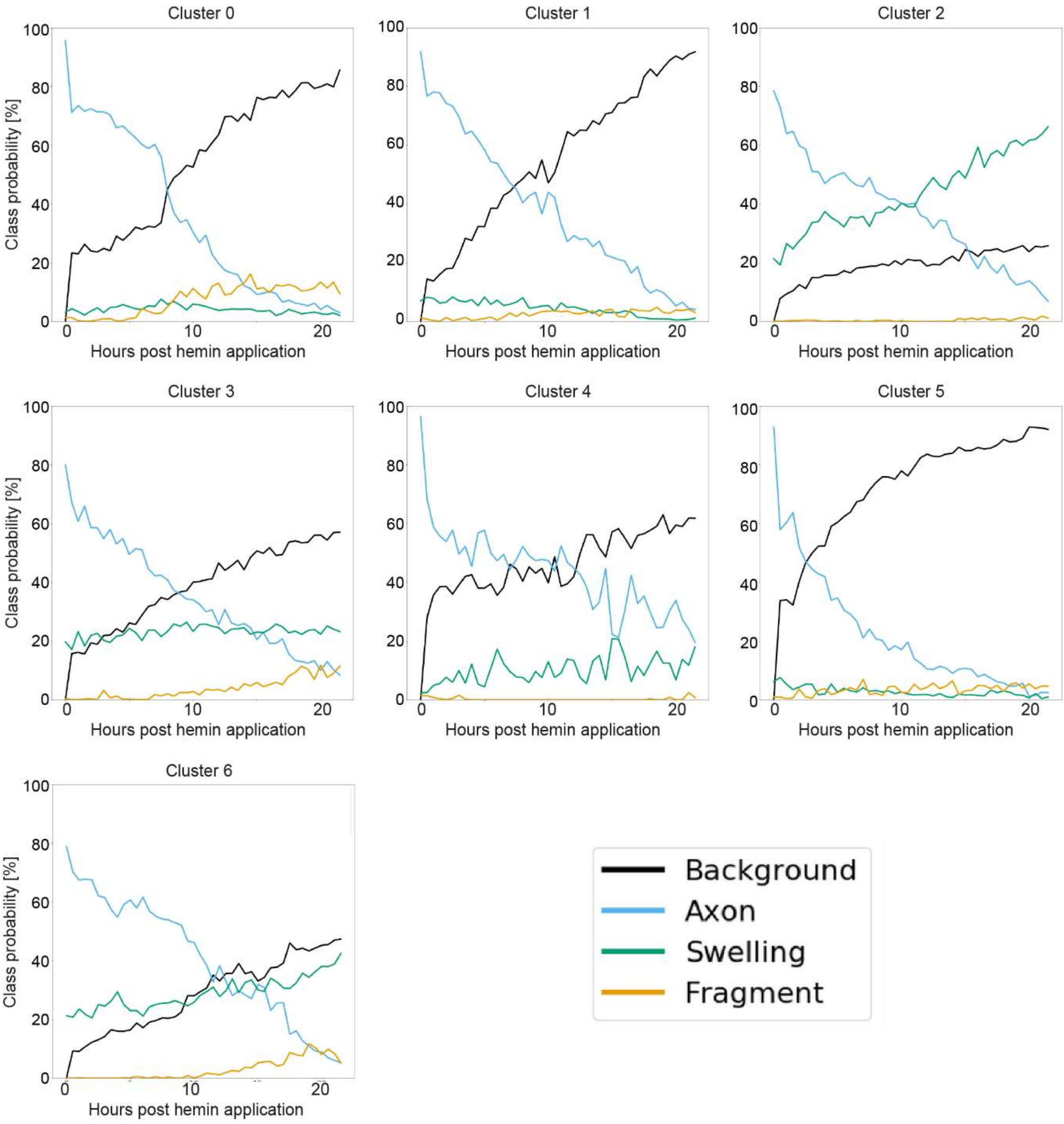

Figure S3. Cluster analysis of the four morphological patterns of AxD based on the changes in class segregation of the pixels. For each time point in comparison to the previous time point, the RNN computed the probability of a change in class for each pixel of an image. Each cluster (0-6) was characterized by an idiosyncratic segmentation pattern over 24 hours.

Table S1. Chemicals and Reagents.

Product	Company	Catalogue number
B-27™ Plus Neuronal Culture System	Thermo Fisher Scientific	A3653401
Boric acid	Merck	925K12283365
Bovin serum albumin	Sigma Aldrich	A9418
Calcein AM	Santa Cruz	sc-203865
Chicken trypsin inhibitor	Sigma Aldrich	T9253
DAPI	Roche	10236276001
DNAse	Sigma Aldrich	D5025-15KU
Earle's balanced salt solution	Sigma Aldrich	E7510
Epoxy solution Smooth-Cast 310/1	KauPo	09202-003-000002
Ethanol 70%	Carl Roth	9065.5
Ethylenediaminetetraacetic acid	Carl Roth	8043.3
Fetal calf serum	Thermo Fisher Scientific	A15-151
Hemin	Sigma Aldrich	H9039
Horse serum	GE Health Life Sciences	SH30074.03
Laminin	Sigma Aldrich	L2020
L-cysteine	Carl Roth	1693.2
MEM, GlutaMAX™ Supplement	Thermo Fisher Scientific	41090028
Papain	Carl Roth	8933.1
Penicillin/Streptomycin	Biochrom	A2212
Phosphate buffered saline	Thermo Fisher Scientific	14200083
Polydimethylsiloxane	Biesterfeld Spezialchemie GmbH	5498840000
Poly-d-lysine	Sigma Aldrich	P6407
Polymethylmethacrylate	Kongsback	Customized
Sodium acetate	Carl Roth	X891.1
Sodium bicarbonate	Sigma Aldrich	S5761
Sodium pyruvate	Thermo Fisher Scientific	11360070
Sodium tetraborate	Merck	1.06308.1000
Triton-X-100	Fluka	93420

Table S2. Antibodies.

**Antibody**	**Company**	**Catalogue number**	**RRID**
Polyclonal rabbit anti-microtubule-associated protein 2 (MAP2)	Abcam	ab32454	AB_776174
Monoclonal mouse anti-synaptophysin	Thermo Fisher Scientific	MA1-213	AB_2723681
Goat anti-mouse IgG (H+L) highly cross-adsorbed secondary antibody, Alexa Fluor 546	Thermo Fisher Scientific	A-11030	AB_2534089
Goat anti-rabbit IgG (H+L) highly cross-adsorbed secondary antibody, Alexa Fluor 488	Thermo Fisher Scientific	A-11034	AB_2576217

Table S3. Time-dependent AxD in an *in vitro* model of hemorrhagic stroke.

**Readout**	**Mauchly test**	**Omnibus Test**	**Posthoc Test**
**Axon area**	[chi]2 is undefined because the number of repeated measurements is greater than the sample size; hence, sphericity was not assumed and the Greenhouse-Geisser correction was applied	one-way ANOVA with Greenhouse-Geisser correction ($\varepsilon = 0.045$) across time*group: $F(6.109) = 19.758$, $P < 0.001$, partial-$\eta^2 = 0.748$	Post hoc Bonferroni $P = 0.018$ for 50 μM from 15 hours ($P < 0.001$ from 19 hours), $P = 0.040$ for 100 μM from 14 hours ($P < 0.001$ from 18.5 hours), $P = 0.020$ for 200 μM from 11.5 hours ($P < 0.001$ from 15 hours) vs. 0 μM
**Axonal swelling area**		one-way ANOVA with Greenhouse-Geisser correction ($\varepsilon = 0.042$) across time*group: $F(5.703) = 3.201$, $P = 0.013$, partial-$\eta^2 = 0.324$	Post hoc Bonferroni $P = 0.030$ for 50 μM from 8 hours, $P = 0.019$ for 100 μM from 6 hours, $P = 0.010$ for 200 μM from 6 hours until 18.5 hours vs. 0 μM
**Axonal fragment area**		one-way ANOVA with Greenhouse-Geisser correction ($\varepsilon = 0.026$) across time*group: $F(3.522) = 9.115$, $P < 0.001$, partial-$\eta^2 = 0.578$	Post hoc Bonferroni $P = 0.044$ for 100 μM from 17 hours, $P = 0.037$ for 200 μM from 9 hours vs. 0 μM

Table S4. AUC analyses of hemin-mediated AxD.

	Kolmogorov-Smirnov test	Levené test	Omnibus Test	Posthoc Test
**Axon**	Z = 0.093, *P* = 0.200	F(3,20) = 0.116, *P* = 0.949	one-way ANOVA F(3,20) = 8.547, *P* = 0.001, partial-η^2 = 0.562	Post hoc Bonferroni *P* = 0.026 for 50 μM, *P* = 0.018 for 100 μM, and *P* < 0.001 for 200 μM vs. 0 μM
**Axonal swelling**	Z = 0.104, *P* = 0.200	F(3,20) = 0.993, *P* = 0.416	one-way ANOVA F(3,20) = 6.721, *P* = 0.003, partial-η^2 = 0.502	Post hoc Bonferroni *P* = 0.012 for 50 μM, *P* = 0.005 for 100 μM, and *P* = 0.016 for 200 μM vs. 0 μM
**Axonal fragment**	Z = 0.245, *P* = 0.001	F(3,20) = 5.800, *P* = 0.005	Kruskal-Wallis Test [chi]2(3,N = 24) = 16.393, *P* = 0.001, η^2 = 0.713	Post hoc Mann-Whitney test with Bonferroni-Holm correction, *P* = 0.037 for 50 μM, *P* = 0.008 for 100 μM and 200 μM vs. 0 μM

Table S5. Comparison of the slopes of the linear regression of the four morphological patterns of AxD in an *in vitro* model of hemorrhagic stroke.

	Comparison of the slopes with Bonferroni-Holm correction for multiple comparisons
**Granular degeneration vs. retraction degeneration**	F(1,44) = 6.971, *P* = 0.034
**Granular degeneration vs. swelling degeneration**	F(1,44) = 0.202, *P* = 1.000
**Granular degeneration vs. transport degeneration**	F(1,44) = 8.865, *P* = 0.026
**Retraction degeneration vs. swelling degeneration**	F(1,44) = 7.842, *P* = 0.030
**Retraction degeneration vs. transport degeneration**	F(1,44) = 0.406, *P* = 1.000
**Swelling degeneration vs. transport degeneration**	F(1,44) = 9.074, *P* = 0.026

Supplementary Movies

Video S1 (separate file). Time-lapse video of axons treated with 0 μM hemin. Primary cortical axons were treated with vehicle (0 μM) for 24 hours and recorded by time-lapse microscopy in a 30-minutes interval and shown in 7 frames/second. Scale bar: 50 μm.

Video S2 (separate file). Time-lapse video of axons treated with 50 μM hemin. Primary cortical axons were treated with 50 μM hemin for 24 hours and recorded by time-lapse microscopy in a 30-minutes interval and shown in 7 frames/second. Scale bar: 50 μm.

Video S3 (separate file). Time-lapse video of axons treated with 100 μM hemin. Primary cortical axons were treated with 100 μM hemin for 24 hours and recorded by time-lapse microscopy in a 30-minutes interval and shown in 7 frames/second. Scale bar: 50 μm.

Video S4 (separate file). Time-lapse video of axons treated with 200 μM hemin. Primary cortical axons were treated with 200 μM hemin for 24 hours and recorded by time-lapse microscopy in a 30-minutes interval and shown in 7 frames/second. Scale bar: 50 μm.

Video S5 (separate file). Time-lapse video of granular degeneration induced by hemin. Granular degeneration of primary cortical axons treated with 200 μM hemin for 24 hours and recorded by time-lapse microscopy in a 30-minutes interval and shown in 7 frames/second. Scale bar: 20 μm.

Video S6 (separate file). Time-lapse video of retraction degeneration induced by hemin. Retraction degeneration of primary cortical axons treated with 200 μM hemin for 24 hours and recorded by time-lapse microscopy in a 30-minutes interval and shown in 7 frames/second. Scale bar: 20 μm.

Video S7 (separate file). Time-lapse video of swelling degeneration induced by hemin. Swelling degeneration of primary cortical axons treated with 200 μM hemin for 24 hours and recorded by time-lapse microscopy in a 30-minutes interval and shown in 7 frames/second. Scale bar: 20 μm.

Video S8 (separate file). Time-lapse video of transport degeneration induced by hemin. Transport degeneration of primary cortical axons treated with 200 μM hemin for 24 hours and recorded by time-lapse microscopy in a 30-minutes interval and shown in 7 frames/second. Scale bar: 20 μm.

Video S9 (separate file). Time-lapse video of the segmentation by the recurrent neuronal networks of AxD induced by hemin. Segmentation of degenerating primary cortical axons treated with 200 μM hemin for 24 hours and recorded by time-lapse microscopy in a 30-minutes interval and shown in 7 frames/second. Scale bar: 100 μm.
